# Dynamic Optical Coherence Tomography: A Non-Invasive Imaging Tool for the Distinction of Nevi and Melanomas

**DOI:** 10.3390/cancers15010020

**Published:** 2022-12-20

**Authors:** Maria Katharina Elisabeth Perwein, Julia Welzel, Nathalie De Carvalho, Giovanni Pellacani, Sandra Schuh

**Affiliations:** 1 Department of Dermatology and Allergology, University Hospital Augsburg, Sauerbruchstraße, 86179 Augsburg, Germany; 2 Department of Dermatology, Federal University of Rio de Janeiro, Avenida Pedro Calmon, Rio de Janeiro 21941-853, Brazil; 3 Department of Dermatology, University of Modena and Reggio Emilia, Via Universita, 41121 Modena, Italy

**Keywords:** dynamic optical coherence tomography, D-OCT, skin microvascularization, unclear melanocytic lesions, melanoma, nevus, dysplastic nevus, non-invasive diagnostics in dermatology, skin imaging, skin cancer screening

## Abstract

**Simple Summary:**

Nevi and melanomas are usually distinguished based on the current gold standard of a clinical-dermoscopic evaluation. Unclear cases, on the other hand, may require additional imaging. Optical coherence tomography (OCT) is a non-invasive imaging technique. It is routinely used for non-melanoma skin cancer but failed to recognize distinguishing features in melanocytic lesions. Dynamic OCT (D-OCT) visualizes microvascularization and has shown promise regarding non-melanoma skin cancer and melanomas. Currently, larger studies on nevi are lacking. Therefore, in this study, we described specific microvascular parameters in nevi and dysplastic nevi, compared them to melanomas, and evaluated D-OCT’s potential for differentiating melanocytic lesions. The addition to the clinical-dermoscopic examination may improve the diagnostic approach to unclear melanocytic lesions, limit unnecessary biopsies, and accelerate and individualize the treatment plan.

**Abstract:**

Along with the rising melanoma incidence in recent decades and bad prognoses resulting from late diagnoses, distinguishing between benign and malignant melanocytic lesions has become essential. Unclear cases may require the aid of non-invasive imaging to reduce unnecessary biopsies. This multicentric, case-control study evaluated the potential of dynamic optical coherence tomography (D-OCT) to identify distinguishing microvascular features in nevi. A total of 167 nevi, including dysplastic ones, on 130 participants of all ages and sexes were examined by D-OCT and dermoscopy with a histological reference. Three blinded analyzers evaluated the lesions. Then, we compared the features to those in 159 melanomas of a prior D-OCT study and determined if a differential diagnosis was possible. We identified specific microvascular features in nevi and a differential diagnosis of melanomas and nevi was achieved with excellent predictive values. We conclude that D-OCT overcomes OCT´s inability to distinguish melanocytic lesions based on its focus on microvascularization. To determine if an addition to the gold standard of a clinical-dermoscopic examination improves the diagnosis of unclear lesions, further studies, including a larger sample of dysplastic nevi and artificial intelligence, should be conducted.

## 1. Introduction

Melanoma is the most aggressive skin tumor, causing over 90% of skin cancer mortality [1]. It is one of the leading cancers in humans [1,2], and its incidence has risen faster than that of most other malignancies [1,3]. The current gold standard of a clinical-dermoscopic examination partly overlooks malignant cases [4] and results in unnecessary biopsies of benign lesions [5]. Distinguishing dysplastic nevi from in situ and early melanomas is challenging and may require the aid of non-invasive imaging [6].

Dermoscopy and confocal laser microscopy (CLM) cannot visualize deeper vascular changes due to a limited penetration depth, and blood vessels are often hidden in pigmented lesions. Tissue shrinking and vessel collapse after fixation limit the diagnostic value of histology [7].

Vascular changes in melanocytic lesions relate to (patho)physiological processes [8,9], and neo-angiogenesis is a hallmark of cancer [10]. Melanomas rely on enlarged, irregular blood vessels to supply their metabolic demands. Hypoxia and tissue architecture play a role in tumor nourishment and oxygen delivery and an increased tumor progression [11,12,13]. Understanding skin homeostasis and early (vascular) changes in malignant transformation may enable earlier detection of malignant changes [14,15,16].

In (D-)OCT (dynamic optical coherence tomography) scans at 1300 nm, melanin is nearly transparent [17], and all lesions, from highly pigmented to amelanotic ones, can be equally well evaluated [18]. Structural OCT correlates well with histological sections [19,20,21] and is routinely used to diagnose non-melanoma skin cancer (NMSC) but cannot distinguish melanocytic lesions. Although a limited resolution was suggested as the cause [22], even high-definition OCT brought no improvement [23,24]. D-OCT, also known as angiographic OCT, is based on the principles of speckle variance OCT [25]. It visualizes the microvasculature in melanocytic lesions [26,27,28] and may answer the demand for a non-invasive, real-time imaging method for unclear melanocytic lesions. According to our findings, microvascular features can distinguish nevi from melanomas.

## 2. Materials and Methods

### 2.1. Participants, Study Course, and Data Management

In this multicentric, prospective, case-control study, subjects with a clinical-dermoscopic suspicion of a nevus or dysplastic nevus were recruited at the University Hospital Augsburg (UKA) and the University of Modena and Reggio Emilia (UNIMORE) between July 2019 and March 2020. Each lesion was scanned by D-OCT and validated histologically. Approval (P30-14, 24 February 2014) was granted by the ethics committee of the Ludwig Maximilian University of Munich. Written informed consent was acquired before study inclusion in all cases. Participant handling was performed lege artis and according to the ethical standards of the Declaration of Helsinki [29]. Data collection and management were conducted in pseudonymized form and according to the legislative acts of the European parliament and council [30].

### 2.2. D-OCT

We used a VivoSight^®^ device with dynamic OCT processing software (Michelson Diagnostics Ltd., Maidstone, Kent, UK) with a Class 1M (EN 60825-1) laser source of near-infrared wavelength (1305 nm) [31]. A total of 120 images with 50 μm spacing are acquired as a 6 × 6 × 2 mm^3^ (width × length × depth) stack. Limited by speckle noise, the best quality dynamic images are achieved down to 500 μm, and the chosen depths (150, 300, and 500 µm) correspond to the epidermal stratum granulosum/spinosum, dermo-epidermal junction (DEJ), and dermal papillary layer.

D-OCT scans were evaluated independently by three blinded and extensively trained analyzers at the UKA and the Federal University of Rio de Janeiro (RIO). Deviations in the scan analyses were discussed, and a consensus sheet was created. We used the OCT-Fitter V2.1 program [32] for visualization and evaluated the scans according to a regime based on the “5D of D-OCT terminology” [33] at three scanning depths and with a total of 117 options per scan. If a vessel shape occurred three times in a scan, it was labeled “present”.

We compared the scans to previously determined reference images to determine parameters such as vessel density and diameter. For a comparison to melanomas, we used scans of our previously conducted melanoma study [18] that had been similarly evaluated and determined the main vascular parameters in nevi and melanomas.

### 2.3. Dermoscopy and Histology

The clinical examination included a description of the lesion course, body location, and palpability. We evaluated the lesion appearance using an ILLUCO IDS-1100 (DermoScan GmbH, Regensburg, Germany) dermatoscope and performed the seven-point checklist. All lesions in both studies were biopsied or excised and assessed by certified histologists after hematoxylin–eosin staining.

### 2.4. Statistical Analysis

The statistical analysis was performed with the help of the UNIKA-T Augsburg and IMBS Lübeck, using the IBM^®^ SPSS^®^ statistics software (SPSS 28.0, IBM Corp., Armonk, NY, USA). The data showed no normal distribution. *p*-values below 0.05 were considered significant, and multiple nevi on one participant were considered independent. For a comparison of microvascularization in nevi and adjacent skin, paired tests were conducted (McNemar test for nominal and Wilcoxon signed-rank test for ordinal data), and in nevi, dysplastic nevi and melanomas, nevus subtypes, and for various influencing factors, unpaired sample tests were performed (Fisher–Freeman–Halton exact test for three and Fisher´s exact test for two samples). We performed binary logistic regression and receiver operating characteristic (ROC) curves with area under the curve (AUC) using the main distinguishing vascular features to determine predictive values, sensitivity, and false positive rates for the differential diagnosis of nevi and melanomas.

## 3. Results

### 3.1. Study Population

Our nevus study included 130 participants with 167 lesions, 84 (50%) on female and 83 (50%) on male participants. The age varied from 9 to 75 years, with a median of 43. Most lesions were located on the back 78 (47%), legs 32 (19%), and abdomen 23 (14%), and fewer on the chest: 17 (10%), arms 8 (5%), head 6 (4%), and palmoplantar 3 (2%). According to histology, 84 (50%) of the lesions were compound, 23 (14%) junctional, 23 (14%) dysplastic, 20 (12%) dermal, 14 (8%) Spitz, and 3 (2%) blue nevi. Regarding melanomas, out of the 159 lesions, 6 (4%) were unclassified, 108 (68%) were superficial spreading, 23 (14%) nodular, 2 (1%) acrolentiginous, and 20 (13%) of the lentigo maligna type. We compared 144 (44%) nevi and 23 (7%) dysplastic nevi of our nevus study to 159 (49%) melanomas of our melanoma study.

### 3.2. Nevi and Adjacent Skin

**Figure 1:**
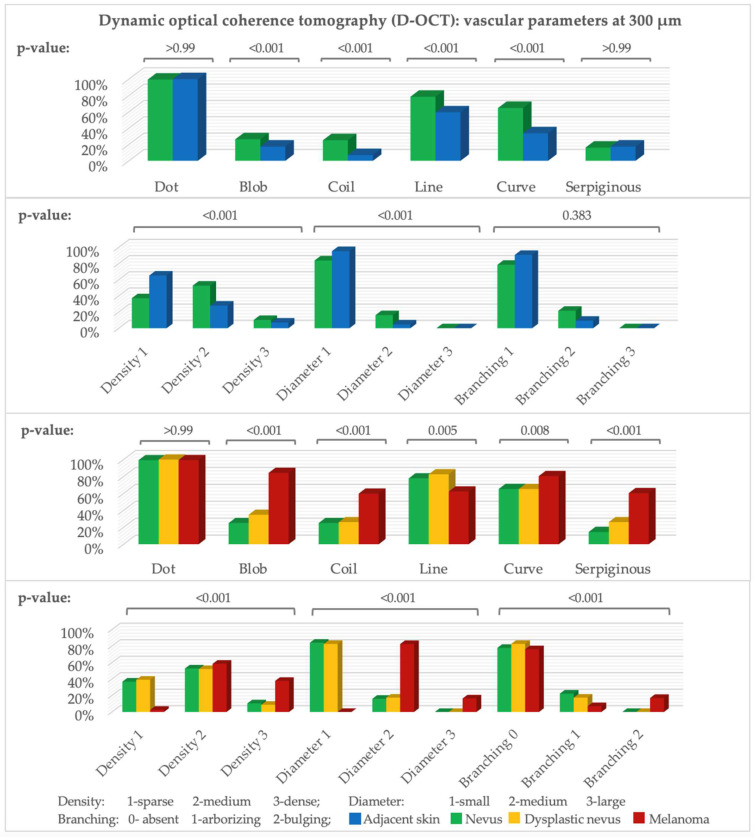
D-OCT vascular parameters. *Y*-axis: number of scans (%), *x*-axis: parameters at 300 µm. Above: *p*-value (McNemar test: nevus–adjacent skin, Fisher–Freeman–Halton exact test: nevi, dysplastic nevi, melanomas). n (adjacent skin = 86, nevus = 144, dysplastic nevus = 23, melanoma = 159).

There are significant differences at all three depths, and they are especially seen at 300 µm. Dots occurred in nearly all scans, and the number of vessels increased with depth (Figure 1). All vessel shapes were more frequent in nevi *(p-value for blobs, coils, lines, and curves at 300 µm and for coils, curves, and serpiginous vessels at 500 µm: <0.001; for dots and serpiginous vessels at 300 µm: >0.99; for blobs at 500 µm: 0.011).* Density *(p-value at 300 and 500 µm: <0.001)* and diameter *(p-value at 300 µm: < 0.001 and 500 µm: 0.002)* were higher in nevi. At 500 µm, a mottled pattern was slightly more common in adjacent skin, and meshes in nevi. Arborizing was insignificantly more frequent in nevi. Bulging branching, cloud pattern, or a specific orientation barely occurred. Nevi showed a rather clustered and adjacent skin a regular or no distribution.

### 3.3. Nevi, Dysplastic Nevi, and Melanomas

Blobs, coils, curves, columns, and serpiginous vessels were significantly more frequent in melanomas *(at 300 µm and 500 µm p-value for blobs, coils, and serpiginous vessels: <0.001 and for curves at 300 µm: 0.008)* and lines *(p-value at 300 µm: 0.005 and at 500 µm: 0.001)* in nevi (Figure 1). Dots occurred in nearly all scans *(p-value at 300 µm: >0.99)*. Blobs, lines, and serpiginous vessels were insignificantly more frequent in dysplastic nevi than nevi *(p-value at 300 µm for blobs: 0.32, lines: 0.79, and serpiginous vessels: 0.22, and for coils and curves: >0.99)*.

**Figure 2:**
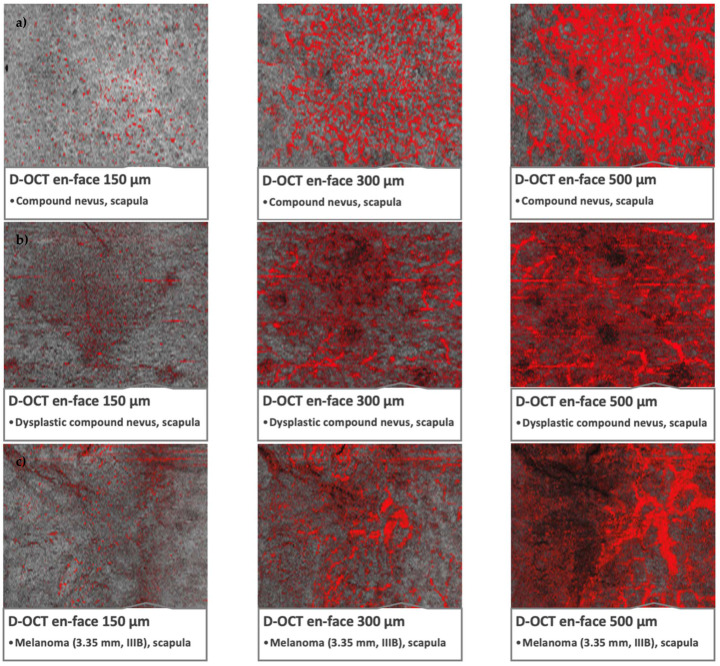
Microvascularization in nevi, dysplastic nevi, and melanomas in D-OCT scans. D-OCT scans (6 × 6 mm^2^). (**a**) Nevus: compound nevus on the scapula with globular appearance and recent changes. (**b**) Dysplastic nevus: flat lesion on the scapula with a combined complex appearance, atypical network, irregular pigmentation, and dots/globules. (**c**) Melanoma: lesion on the scapula, tumor thickness 3.35 mm, pT3aN1bM0S2, stage IIIB.

Density and diameter in melanomas were significantly higher *(p-value at 300 µm: <0.001)*. In nevi, small diameter and sparse to medium density prevailed. Unlike nevi and dysplastic nevi, melanomas showed bulging rather than arborizing branching *(p-value at 300 µm: <0.001)*. The vessel distribution in melanomas was significantly more often irregular, while it was regular or clustered in the other groups (Figure 1 and Figure 2).

In the next step, we screened for major distinguishing features (blobs, coils, lines, serpiginous vessels, density, and diameter) between nevi (excluding dysplastic lesions) and melanomas and evaluated their diagnostic value by binary logistic regression. Individual parameters often showed no significance, but altogether, according to these parameters, melanomas and nevi at 300 µm depth were recognized with predictive values of 96.8% and 88.2%, respectively, while at 500 µm, the predictive values were 95.5% and 91.0% (Table 1).

**Figure 3:**
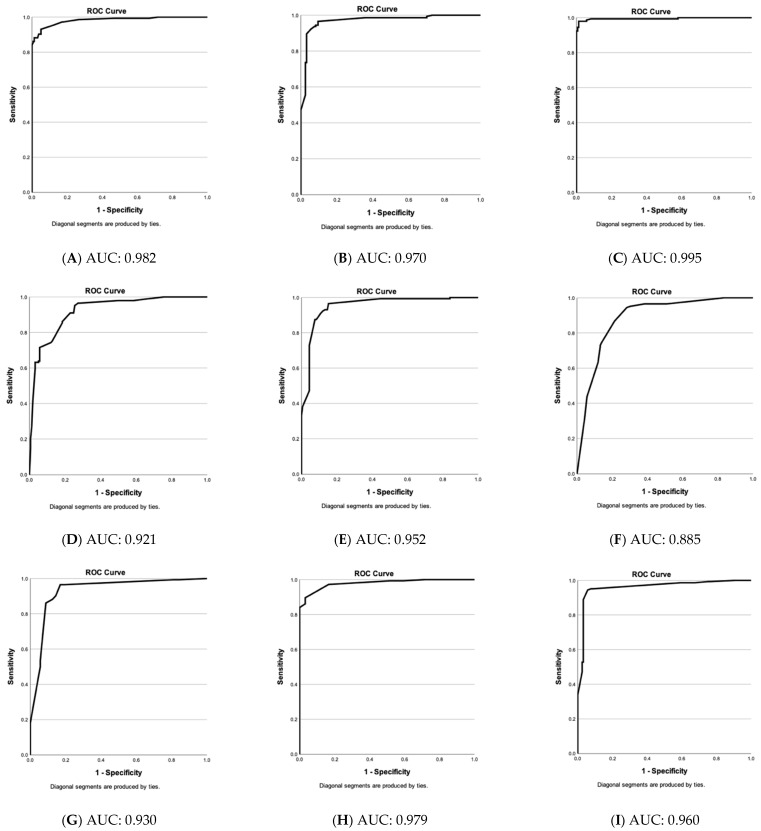

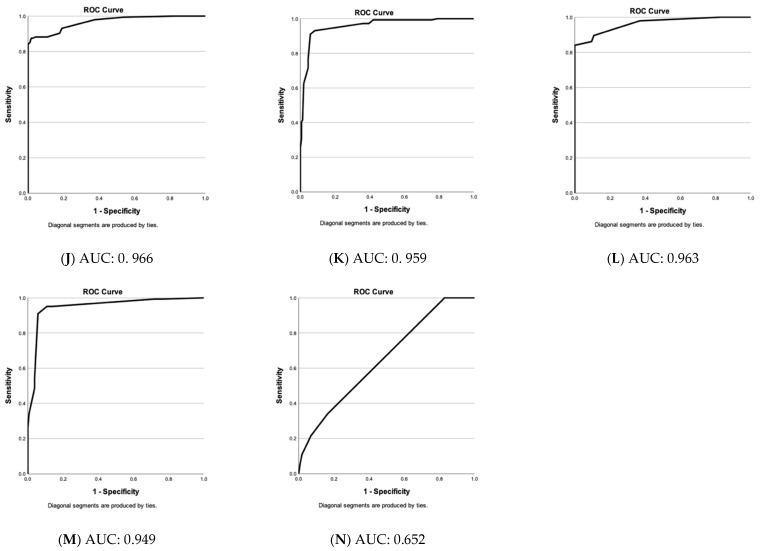
Evaluation of vascular parameter choices in the differential diagnosis of nevi & melanomas. Receiver operating characteristic (ROC)—curves of predicted values (nevi versus melanomas) by binary logistic regression based on specific vascular parameter patterns (**A**–**N**). Y-axis: sensitivity of test and y-axis: 1—specificity. Below: area under the curve (AUC) value. (**A**) Blob, coil, line, serpiginous, density & diameter at 300 µm. (**B**) Blob, coil, line, serpiginous, density & diameter at 500 µm. (**C**) Blob, coil, line, serpiginous, density & diameter at 300 & 500 µm. (**D**) Blob, coil, line, serpiginous & density at 300 µm. (**E**) Blob, coil, line, serpiginous & density at 500 µm. (**F**) Blob, coil, line, serpiginous at 300 µm. (**G**) Blob, coil, line, serpiginous at 500 µm. (**H**) Blob, line, serpiginous & diameter at 300 µm. (**I**) Blob, line, serpiginous & diameter at 500 µm. (**J**) Blob, serpiginous, density & diameter at 300 µm. (**K**) Blob, serpiginous, density & diameter at 500 µm. (**L**) Blob, serpiginous & diameter at 300 µm. (**M**) Blob, serpiginous & diameter at 500 µm. (**N**) Dot, blob, coil, line & serpiginous at 150 µm (non-significant vascular parameters at 150 µm).

Figure 3 stresses that the choices of vascular parameters (A–M) have a very high sensitivity and low false positive rate. Only vascular parameters showing significant differences between nevi and melanomas were included except for (N), where dot, blob, coil, line, and serpiginous vessel at 150 µm depth are shown as examples of an “unsuitable” choice of parameters.

Figure 3 stresses that the choices of vascular parameters (A–M) have a very high sensitivity and a low false positive rate. Only parameters with significant differences between nevi and melanomas were included except for (N), where an example of an “unsuitable” choice of parameters is shown. With an AUC of 0.995, the “blob, coil, line, serpiginous, density, and diameter at 300 and 500 µm-group” results in the best result and is followed by “blob, coil, line, serpiginous, density, and diameter at 300 µm” (0.982) and “−500 µm” (0.970). If no computerized evaluation is conducted, these groups include too many parameters for a routine scan analysis. Therefore, we compared groups with fewer parameters. Vessel diameter proved more important than density and coils (with coils, excluding density and diameter: (F) at 300 µm: 0.885 and (G) at 500 µm: 0.930; with density and coils, without diameter: (D) at 300 µm: 0.921 and (E) at 500 µm: 0.952; with diameter, without density and coils: (H) at 300 µm: 0.979 and (I) at 500 µm: 0.960).

In summary, the parameters, blob, serpiginous vessel, density, and diameter at 300 µm depth seem most reliable for the differential diagnosis.

### 3.4. Nevus Subtypes

Serpiginous vessels at 500 µm were most frequent in dysplastic and least common in junctional and dermal *nevi (p-value 0.046)*. Blobs at 300 µm were most often observed in Spitz, dysplastic, and dermal nevi and least often in junctional ones *(p-value: 0.01)*. Spikes and columns were mostly seen in Spitz and least frequently in junctional nevi *(p-value for spikes: 0.005 and columns: 0.17)*. Density and diameter did not differ except for a higher density at 500 µm in Spitz and dysplastic nevi and a lower one in junctional nevi *(p-value: 0.004).*

### 3.5. Potential Influencing Factors

**Figure 4:**
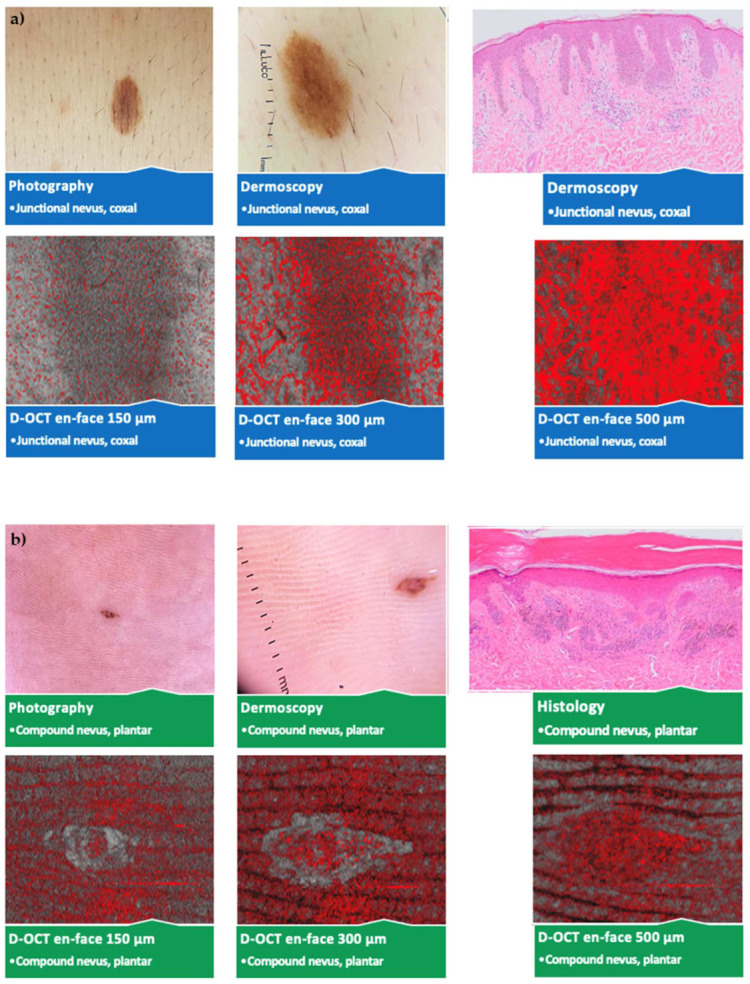

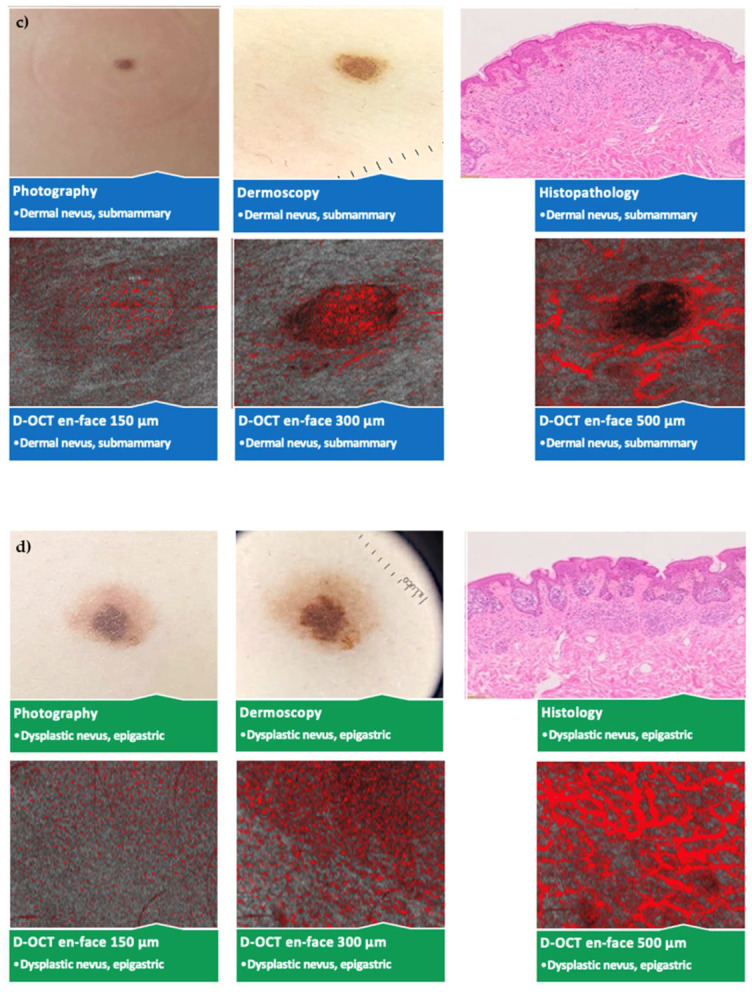

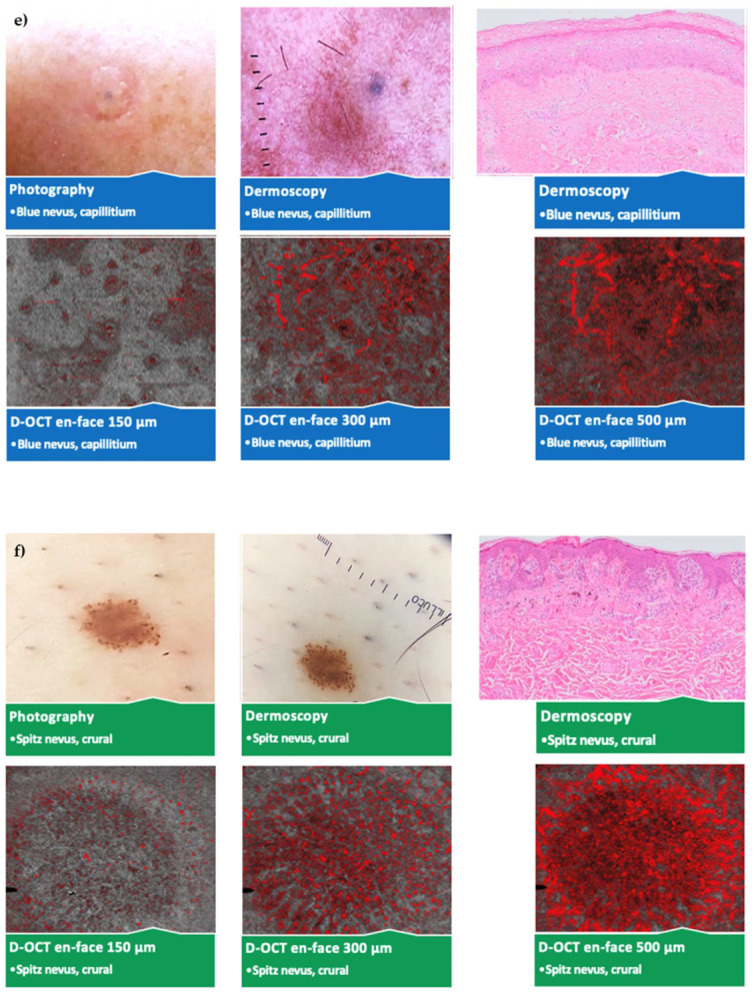
Comparison of different nevus subtypes and lesion locations. Photography, dermoscopy (10 × 10 mm^2^); D-OCT (6 × 6 mm^2^) scans at 150, 300, and 500 µm, and histological (Leica DM LB2) sections; (**a**) junctional nevus on the hip; (**b**) compound nevus on the sole of the foot; (**c**) dermal nevus below the mamma; (**d**) dysplastic nevus in the epigastrium; (**e**) blue nevus on the capillitium; (**f**) Spitz nevus on the lower leg.

No significant vascular differences occurred between different sexes and ages except for more spikes and blobs at 500 µm in young (<40 years) participants. Several statistically significant differences according to the lesion location without a pattern were observed. An overview of the photography, dermoscopy, and D-OCT findings in different nevus subtypes and lesion locations is shown in Figure 4.

### 3.6. Clinical, Dermoscopic, and Histological Evaluation

A total of 88 of 167 nevi were flat, 62 just palpable, and 17 papular-nodular. A total of 48 (29%) showed a seven-point-checklist score of 3 or more. Based on a clinical and dermoscopic examination, 59 lesions were suspected to be benign nevi, 81 dysplastic, 20 melanomas, 5 melanomas or Spitz nevi, and 2 basal cell carcinomas. While pre-histologically, 108 (65%) of lesions were considered malignant, dysplastic, or unclear, histologically, only 23 (14%) proved to be dysplastic and none malignant.

## 4. Discussion

Early recognition and treatment are the most efficient way to improve melanoma prognosis [34,35,36]. An example is a decrease in melanoma mortality due to a clinical-dermoscopic examination in the German skin cancer screening program [37].

OCT correlates well with histological sections [19,20,21,38], and it is routinely used to diagnose NMSC. Still, neither conventional nor high-definition OCT [23,24] can visualize distinguishing features in melanocytic lesions [23,24]. D-OCT shows physiological blood flow changes [9], influences of topical treatments [39], and in vivo microvascularization in tumors, inflammatory, and degenerative dermatological conditions [22,40].

Due to D-OCT´s real-time approach, allowing an immediate generation of a treatment plan or excision of malignant lesions, the diagnostic and therapeutic approach is accelerated and individualized [41]. In addition, D-OCT is suitable for monitoring changes during watch-and-wait approaches [42,43] and follow-ups after tumor resection [44]. Also possible are tumor border control [45,46,47] and staging [18] with a risk stratification, which should be based on vessel morphology, tumor thickness, and risk factors, including atypical vessel patterns.

Because microvascular changes according to D-OCT were observed already in in situ melanomas, angiogenesis seems to occur early in tumor progression and invasion [18]. In thick, deeply invasive melanomas, bizarre vessels with aneurysms appeared at higher depths that could result from a fast, uncoordinated tumor growth [22]. A correlation of the number of atypical vessels to the melanoma thickness according to the Breslow index [27] and a correlation of the thickness and ulceration status with the metastatic status were detected [18]. Since tumor thickness and ulceration status correlated with melanomas with hematogenous rather than lymphatic metastasis, potential as a biomarker of neo-angiogenesis and tumor aggressiveness has been suggested [18].

While there is no lack of D-OCT studies on microvascularization in melanomas, little is currently known about nevi. In our study, melanocytic nevi showed a significantly lower vessel density and diameter, more lines, and fewer blobs, coils, curves, and serpiginous vessels than melanomas. Blobs are immature vessels and coils, and serpiginous vessels are larger, corkscrew and snake-like vessels enabling a higher blood supply than smaller vessels such as dots or lines. These vessel types are essential for and the result of the fast growth, angiogenesis, and high metabolic demands of melanomas and are not frequent in nevi. While nevi showed a somewhat mixed distribution (regular, irregular, or clustered), melanomas had a mostly irregular one, corresponding to the observation of irregular, bizarre vessels in dermoscopy.

Not only a dermoscopic resemblance exists between melanomas and Spitz nevi. D-OCT presented similar features, including more blobs, coils, serpiginous vessels, spikes, and columns in Spitz nevi than in other subtypes. Serpiginous vessels occurred most often in dysplastic nevi and least frequently in junctional nevi. An explanation may be that larger serpiginous vessels occur only at deeper levels and junctional nevi show distinguishing features around the DEJ. Still, distinguishing different nevus subtypes only by D-OCT was not possible.

Internal and external factors influence skin morphology and vascularization [48,49,50,51,52,53,54,55,56,57]. Variations may occur due to intraindividual (lesion location with specific epidermal thickness) and interindividual (age, sex, degree of photodamage, and skin hydration) differences. Aging is connected to impaired microvascular endothelial function. It has been suggested that the blood flow [58] and capillary loop density decrease, and vascular length increases with age [48]. The only significant difference in our study was the occurrence of blobs and spikes, immature and not fully developed vessels, in younger participants (<20 years). According to Hodges et al., females’ resting cutaneous blood flux is lower [57]. However, we did not find differences between the sexes.

Further, variations in lesion microvascularization according to the body area have been suggested [59,60]. This study examined healthy skin adjacent to lesions as a reference. The epidermal thickness may influence the depth of occurring vessels since it is avascular. D-OCT was confirmed to function on all body sites [61], and vascular changes due to physiological influences in different body areas can be visualized [9]. Nevertheless, we did not identify vascular features according to which nevi on different body areas could be distinguished.

In this study, nevi were distinguished from melanomas with excellent predictive values and sensitivity. As the most suitable distinguishing features, we consider the following vascular parameters: blobs, coils, lines, serpiginous vessels, density, and diameter at 300 and 500 µm depth. For the differential diagnosis of nevi and melanomas, at least blobs, serpiginous vessels, density, and diameter should be analyzed in scans.

Based on the clinical-dermoscopic evaluation, as in the clinical practice, the number of dysplastic lesions was clearly overestimated in this study. A D-OCT application has been stated to reduce the lesion number needed to be excised to diagnose a melanoma [62]. In our study, at least 16% of unnecessary excisions (27 lesions with clinical-dermoscopic suspicion of malignancy out of 167) could have possibly been avoided by an additional D-OCT evaluation.

Limitations to this study are the partial subjectivity of D-OCT scan evaluation despite extensive training and the use of reference images, and the number of included dysplastic nevi. Further, the simplified classification system of lesion locations, the lack of participants of dark skin types, and the small number of blue nevi, pediatric, head, and acral lesions.

For clinical work, it is essential to distinguish nevi from dysplastic nevi and dysplastic lesions from melanomas. Therefore, further multicentric studies with larger sample numbers evaluating the potential for distinguishing these lesions and different degrees of dysplasia are required. The integration of artificial intelligence and deep learning is highly recommended to confirm these promising results and to ease and standardize the scan evaluation process.

Other future aims include setting a measure of proficiency regarding scan evaluation, generation of software that automatically recognizes and outlines lesions, and integration in terms of teledermatology.

## 5. Conclusions

We identified specific microvascular parameters in nevi that enable a distinction from melanomas with excellent predictive values and sensitivity, and according to which a substantial number of unnecessary excisions in the daily clinical practice could possibly be prevented. These include blobs, coils, and serpiginous vessels, which are typical for melanomas, and lines in nevi, as well as density and diameter, which are smaller in benign lesions. In addition, D-OCT showed significant differences in nevus microvascularization regarding participant age, lesion location, and subtype but did not allow a differential diagnosis solely based on this method. No differences according to sex were observed.

We conclude that D-OCT can distinguish nevi and melanomas based on microvascularization and may be a valuable addition to the current clinical-dermoscopic gold standard, but further studies evaluating the potential for distinguishing melanomas and dysplastic nevi are required, and the integration of artificial intelligence in a standardized and computerized analysis is recommended.

## Figures and Tables

**Table 1:** Binary logistic regression and classification table of vascular parameters in nevi and melanomas. Major distinguishing features: the presence of blobs, coils, lines, and serpiginous vessels, as well as density and diameter; n (melanoma = 157, nevus = 144); (a) at 300 µm; (b) at 500 µm.

Binary Logistic Regression: 300 µm					Binary Logistic Regression: 500 µm				
		Significance	95% confidence interval				Significance	95% confidence interval	
			Lower	Upper				Lower	Upper
1_blob300(1)		0.035	0.054	0.902	1_blob500(1)		<0.001	0.004	0.036
1_coil300(1)		0.760	0.355	4.129	1_coil500(1)		0.058	0.044	1.052
1_line300(1)		0.003	3.186	262.411	1_line500(1)		<0.001	6.877	367.370
1_serpiginous300(1)		0.031	0.106	0.900	1_serpiginous500(1)		0.021	0.076	0.808
1_density300(1)		0.141			1_density500(1)		0.172		
1_density300(2)		0.066	0.029	1.121	1_density500(2)		0.549	0.139	41.258
1_density300(3)		0.290	0.042	2.570	1_density500(3)		0.915	0.048	15.243
1_diameter300(1)		1.000			1_diameter500(1)		<0.001		
1_diameter300(2)		0.995	0.000	.	1_diameter500(2)		0.009	0.004	0.459
1_diameter300(3)		0996	0.000	.	1_diameter500(3)		<0.001	0.000	0.018
Constant		0.995			Constant		0.004		
**Classification Table ^a^**					**Classification Table ^a^**				
		Predicted					Predicted		
			Study	Percentage Correct				Study	Percentage Correct
Observed		Melanoma	Nevus		Observed		Melanoma	Nevus	
Study	Melanoma	152	5	96.8	Study	Melanoma	150	7	95.5
	Nevus	17	127	88.2		Nevus	13	131	91.0
Overall Percentage				92.7	Overall Percentage				92.7

^a^ The cut value is 0.500.

## Data Availability

Pseudonymized data are available on request.
